# Outcomes of an International Workshop on Preconception Expanded Carrier Screening: Some Considerations for Governments

**DOI:** 10.3389/fpubh.2017.00025

**Published:** 2017-02-24

**Authors:** Caron M. Molster, Karla Lister, Selina Metternick-Jones, Gareth Baynam, Angus John Clarke, Volker Straub, Hugh J. S. Dawkins, Nigel Laing

**Affiliations:** ^1^Office of Population Health Genomics, Public Health Division, Department of Health Western Australia, Perth, WA, Australia; ^2^Sir Charles Gairdner Hospital, Perth, WA, Australia; ^3^Genetic Services WA, Perth, WA, Australia; ^4^School of Paediatrics and Child Health, University of Western Australia, Perth, WA, Australia; ^5^Institute for Immunology and Infectious Diseases, Murdoch University, Perth, WA, Australia; ^6^Telethon Kids Institute, University of Western Australia, Perth, WA, Australia; ^7^Western Australian Register of Developmental Anomalies, Perth, WA, Australia; ^8^Spatial Sciences, Department of Science and Engineering, Curtin University, Perth, WA, Australia; ^9^Division of Cancer and Genetics, School of Medicine, Cardiff University, Cardiff, UK; ^10^Institute of Human Genetics, University of Newcastle upon Tyne, Newcastle upon Tyne, UK; ^11^Centre for Comparative Genomics, Murdoch University, Perth, WA, Australia; ^12^Centre for Population Health Research, Curtin University, Perth, WA, Australia; ^13^School of Pathology and Laboratory Medicine, University of Western Australia, Perth, WA, Australia; ^14^Centre for Medical Research, Harry Perkins Institute of Medical Research, University of Western Australia, Perth, WA, Australia; ^15^Neurogenetics Unit, Department of Diagnostic Genomics, PathWest Laboratory Medicine, Department of Health Western Australia, Perth, WA, Australia

**Keywords:** carrier screening, expanded carrier screening, genetic carrier screening, government, public policy

## Abstract

**Background:**

Consideration of expanded carrier screening has become an emerging issue for governments. However, traditional criteria for decision-making regarding screening programs do not incorporate all the issues relevant to expanded carrier screening. Further, there is a lack of consistent guidance in the literature regarding the development of appropriate criteria for government assessment of expanded carrier screening. Given this, a workshop was held to identify key public policy issues related to preconception expanded carrier screening, which governments should consider when deciding whether to publicly fund such programs.

**Methods:**

In June 2015, a satellite workshop was held at the European Society of Human Genetics Conference. It was structured around two design features: (1) the provision of information from a range of perspectives and (2) small group deliberations on the key issues that governments need to consider and the benefits, risks, and challenges of implementing publicly funded whole-population preconception carrier screening.

**Results:**

Forty-one international experts attended the workshop. The deliberations centered primarily on the conditions to be tested and the elements of the screening program itself. Participants expected only severe conditions to be screened but were concerned about the lack of a consensus definition of “severe.” Issues raised regarding the screening program included the purpose, benefits, harms, target population, program acceptability, components of a program, and economic evaluation. Participants also made arguments for consideration of the accuracy of screening tests.

**Conclusion:**

A wide range of issues require careful consideration by governments that want to assess expanded carrier screening. Traditional criteria for government decision-making regarding screening programs are not a “best fit” for expanded carrier screening and new models of decision-making with appropriate criteria are required. There is a need to define what a “severe” condition is, to build evidence regarding the reliability and accuracy of screening tests, to consider the equitable availability and downstream effects on and costs of follow-up interventions for those identified as carriers, and to explore the ways in which the components of a screening program would be impacted by unique features of expanded carrier screening.

## Introduction

Population-based screening programs are a public health approach implemented by many governments, which usually focus on a specific subpopulation defined by age, sex, and sometimes by ethnicity. Examples include newborn screening, prenatal screening, and screening for breast, bowel, and cervical cancer. In most countries, programs for screening the population to identify carriers of genetic diseases have not yet been adopted by governments. However, the possibility of offering such programs has become more salient in recent years in the wake of technology drivers such as the availability of relatively low cost massively parallel sequencing. Given this, a workshop was held with experts from a number of countries to identify key public policy issues related to preconception expanded carrier screening, which governments should consider before deciding whether to publicly fund such programs.

Carrier screening is a form of genetic testing that is used to determine a couple’s risk of having a child with a recessive genetic disorder, when there is no *a priori* risk based on personal or family history (1). The process involves analyzing a sample of blood, or other biological material, for evidence of genetic mutations associated with autosomal-recessive and X-linked conditions. Carriers of autosomal-recessive conditions are people who have one copy of a gene mutation that can cause a condition in their offspring. If two carriers of a mutation in the same gene have children, their offspring have a one in four chance of having the condition. For women who carry a gene mutation associated with an X-linked condition, their children have a 50% chance of inheriting that gene mutation. Male children of these women are usually affected by the condition since they inherit their only copy of the X chromosome from their mother, while female children are usually protected from the condition by the inheritance of a second X chromosome from the father.

The members of a couple may undertake carrier screening simultaneously or sequentially, and screening can be performed in the preconception period or during pregnancy. If offered in the preconception period, carrier screening provides an opportunity to identify individuals who are at risk of having a child affected with a condition, before they become pregnant. Partners can then make informed reproductive choices including: not having a child at all; adoption; preimplantation genetic diagnosis (PGD) or *in vitro* fertilization (IVF) to avoid having an affected child; or to have a child naturally, with prior knowledge of their risk of having a child with a specific condition. Screening during the preconception period can be considered more favorable than during pregnancy, as it avoids expectant parents being faced with a prenatal diagnosis and possibly a decision on whether to selectively terminate an affected pregnancy as the only way of avoiding the birth of an affected child.

Until recently, carrier screening was generally available for one or a very small number of conditions within ethnic subgroups of the population that have a relatively high prevalence of those conditions. Examples include carrier screening offered to the Ashkenazi Jewish population for Tay–Sachs disease and to Mediterranean populations for beta-thalassemia (2–4). In more recent years, the possibility of expanded carrier screening has emerged. This involves simultaneously screening for carrier status for multiple diseases, which can be offered to all members of a pan-ethnic population, regardless of family history or ancestry (5–7). This has been made feasible through advances in genotyping and genetic sequencing technologies, which enable the concurrent evaluation of genetic mutations for large numbers of recessive diseases, for relatively low additional cost (5). Commercial companies have already developed expanded carrier screening tests that can screen for more than 100 recessive diseases at one time and these are being offered direct-to-consumers at a cost (8, 9). However, only consumers who are willing and can afford to pay for these screening tests are able to undertake them.

For carrier screening to be truly universal, it requires a publicly funded approach to ensure equity of access. To warrant public funding, there needs to be an evidence-based assessment of the appropriateness of expanded carrier screening against a range of predetermined criteria (10). This is because, like all population-based screening programs, carrier screening has the potential to result in harm as well as benefits (11). Therefore, there must be a rigorous assessment before implementing a publicly funded program, to ensure that the benefits outweigh the harms. The “gold standard” criteria for evaluating population-based screening were developed for the World Health Organization over 40 years ago by Wilson and Jungner (12, 13). These screening pioneers suggested assessing evidence against 10 principles that explore four themes: (1) the condition being screened, (2) the test, (3) the treatment, and (4) the screening program.

While the Wilson and Jungner principles are the benchmark for government decision-making in screening, the ways in which they have been applied in practice vary across the globe. This is highlighted through a recent review of the criteria for deciding whether to introduce screening programs in Australia, Canada, Denmark, Finland, France, Germany, Italy, the Netherlands, New Zealand, Sweden, the UK, and the USA (14). Across these countries, Seedat et al. (14) identified 46 unique criteria that were associated with screening in general, most of which related to the screening program (27) as opposed to the condition (7), test (6), and treatment (6). Generally, the reason for expansion beyond the original Wilson and Jungner principles and variation in government decision-making criteria is to ensure processes sufficiently explore the issues most pertinent to each local setting (15, 16).

Despite their continued application to the assessment of screening programs worldwide, the Wilson and Jungner principles do not incorporate the full range of considerations for expanded carrier screening. A key limitation is that the criteria were developed without specific examination of the unique benefits, risks and harms that accompany genetic screening (10). These unique features include that most conditions screened for will be rare and that a genetic test is required that in most cases will produce personal information for both the individual having the test, as well as their genetic relatives. Further, in relation to carrier screening, it does not screen for the presence of a condition, but rather for the presence of gene mutations that might cause a condition in offspring, and the “treatment” following on from carrier screening is thus not an intervention in line with the classic definition. This latter point means that, should an individual be identified as a carrier, there is no treatment required since carriers are generally not affected by the condition for which they are a carrier. Instead, carriers are provided with information that will inform their reproductive choices.

While it is recognized that the Wilson and Jungner principles need further consideration in the context of expanded carrier screening, the Netherlands is so far the only country to have developed criteria specifically for assessing genetic screening including carrier screening (17). For other governments looking to develop relevant criteria, there is a lack of clear, consistent guidance in the literature. At the time our workshop was held, there were two statements of recommendations from professional bodies in the USA regarding expanded carrier screening along with a report by the UK Human Genetics Commission, and recommendations have subsequently been published by the European Society of Human Genetics (ESHG) (1, 7, 18, 19). However, the content of these documents varies considerably, highlighting the current lack of consensus. There is literature that has identified lessons learned and factors for the successful implementation of existing, usually ethnicity-based, carrier screening programs (20–23). Yet, to our knowledge, there has been no systematic evaluation of the extent to which these factors can inform decision-making criteria for assessing expanded carrier screening.

Given the lack of clear, consistent policy and academic guidance on the relevant criteria to assess expanded carrier screening, we believe there is a need for more research to inform governments of the issues they need to consider before implementing expanded carrier screening. In the first instance, best practice public policy development suggests that there is a need to understand the values, expectations, preferences, and concerns of key stakeholders (10). In line with this, we held an international workshop to gain an understanding of which issues experts considered were the most salient for governments to consider. We chose to focus on screening in the preconception period since this is considered to be the best timing for carrier screening to optimize reproductive choice (1, 24).

## Method

In June 2015, we held a satellite workshop at the ESHG Conference. To reach experts who might want to attend the workshop, a call for expressions of interest was posted on the ESHG Conference website. Invitations were also issued to known experts in fields related to expanded carrier screening, with a request to forward the invitation to other experts who might also be interested in the workshop. These communications included information on the objectives of the workshop. One of the objectives listed was “to contribute to the academic literature on expanded carrier screening,” which would be by publishing the outcomes of the workshop in a peer-reviewed journal. The intention to contribute to the academic literature was reiterated in material sent to those who expressed an interest in attending the workshop, as well as at the beginning of the workshop while “setting the scene” and at the end of the workshop in relation to “next steps.” By choosing to participate in the workshop, which was a public event, we assumed that participants were giving implied consent to the workshop outcomes being collated, analyzed, and published in academic literature. We considered this a sufficient level of consent, since there would be no identifying information published about the participants and the information obtained from the workshop was neither personal nor private, and could not be linked to an individual but rather were the outcomes of group discussions in a public setting.

The aim of the workshop was to identify expert opinions on the issues that governments should consider when deciding whether or not to implement preconception expanded carrier screening. To achieve this, the workshop was structured around two design features, these being information provision and small group deliberations. The morning session of the workshop involved a series of presentations from nine experts in different fields relevant to expanded carrier screening. These presentations were designed to expose workshop participants to information from outside their field of expertise and to a range of different perspectives on the key issues that government policymakers might face in relation to preconception expanded carrier screening (see Table 1). Providing a range of perspectives was considered important as it was recognized that these presentations would likely frame the subsequent deliberations of the small groups.

**Table 1:** **Range of perspectives covered in workshop presentations**.

Role	Focus of presentation
Clinical geneticist	Justifications for offering carrier screening, criteria for assessing public health screening, tensions in the goals of carrier screening, different social and cultural contexts in which screening can be offered, and queries regarding the conditions to screen
Screening policymaker	Criteria that guide government decision-making for population-based screening programs, including the Wilson and Jungner principles, and how these could be applied to preconception expanded carrier screening. Issues such as benefits and harms, public support, understanding the condition, testing, feasibility, and cost
Carrier screening program manager	Population and genetic conditions in Israel, national carrier screening programs, carrier rates in the population
Health economist	How health economics is used in decision-making processes, health technology assessment including cost–benefit analysis and cost-effectiveness, types of healthcare costs, and the kinds of health economic questions that arise in the context of developing screening programs
Health consumer advocate	What conditions to screen, benefits, challenges related to infrastructure, awareness, education, and engaging people
Ethicist	Commercial offers of expanded carrier screening and the range of conditions tested for, criteria to determine “severe” conditions, reproductive decision-making, individual, and social impact
Laboratory scientist	Carrier screening recommendations by professional bodies, test characteristics such as clinical utility and validity and analytic validity, technology that enables expanded carrier screening, condition/mutation selection, pathogenic variants detected and variants of uncertain significance, carrier frequency, education, and counseling
Disability rights advocate	Disability rights objections to expanded carrier screening, reproductive decisions after carrier screening, eugenics, *in vitro* fertilization, discrimination, condition selection
Clinical geneticist	The evolution of reproductive carrier screening, counseling, and education in the past and for expanded carrier screening

Following the presentations, participants worked in small groups of between six and eight people to discuss and develop answers to three questions, namely:

1. What are the key factors/issues that governments need to consider when deciding whether or not to implement publicly funded, whole-population preconception carrier screening programs?
2. What are the benefits of implementing publicly funded whole-population preconception carrier screening programs?
3. What are the risks and challenges of implementing publicly funded whole-population preconception carrier screening programs?

The outcomes for each small group were written down by a scribe on feedback sheets and then orally reported back to the large group. The information recorded by the scribes was subsequently analyzed to identify common themes across all groups, and a summary of these findings is included in this paper.

## Results

Forty-one people attended the workshop, representing a range of disciplines including human genetics, clinical genetics, medical genetics, genetic counseling, primary care, pediatrics, laboratory science, bioethics, population health policy, medical sociology, humanities, health economics, and public health genomics. Participants were largely employed in academia, public health systems, and commercial companies. The outcomes of the small group discussions are presented below, gathered together in line with the Wilson and Jungner themes of the condition, test, treatment, and screening program.

### Condition

There was general agreement that only “severe” or “serious” conditions should be included in preconception expanded carrier screening. However, there was concern about the lack of a consensus definition of “severe” and “serious,” where the line between “mild” and “severe” should be and why. Some participants suggested “severe” disorders should be defined as early onset conditions where the child dies in the newborn or early childhood periods. There was a belief that screening individuals in line with this definition is (1) less ethically contentious than screening for conditions that do not result in early mortality; (2) avoids perceptions of eugenics; (3) has fewer implications for people living with diseases; and (4) is less vulnerable to the disability rights critique that carrier screening removes normal human diversity.

The lack of a definition of severity was perceived to create confusion regarding which conditions should be screened and the potential for competition between laboratories to offer more and more tests. There was a belief that commercial pressures and technology-led development of expanded carrier screening have the potential to result in a “slippery slope” of offering tests just because they are possible. Thus, participants perceived that a definition of severity, which could be used to determined which conditions to screen, may safeguard against the inappropriate extension of preconception expanded carrier screening programs to include more and more conditions, including “mild” conditions. Another question raised at the workshop was whether participants of a preconception expanded carrier screening program should be able to choose which conditions to be tested for, or whether they could be offered a panel with no option for selecting specific conditions to be tested for.

### Test

Participants argued that there is a need for robust up-to-date evidence about tests used in preconception expanded carrier screening. Specifically, evidence is needed on the following: the reliability of the tests (especially the negative predictive value) and their appropriateness for the population; the confidence with which the pathogenicity of the gene mutations has been established; clinical and analytical validity; and residual risk and explanations for variants of unknown significance. It was thought that the tests should have clinical value/utility and public acceptability, and economic factors including the cost of tests need to be considered in deciding which conditions to test for.

### Treatment

There were no issues raised in relation to “the treatment” of participants identified as carriers. This undoubtedly relates to the fact that carriers of autosomal-recessive diseases are unaffected by those diseases, and as such they do not need treatment. Carriers of X-linked diseases may sometimes be or become affected, depending upon multiple factors including the pattern of X chromosome inactivation.

### Screening Program

For participants, there were many uncertainties around preconception expanded carrier screening and the view that offering a program would be “uncharted waters” in the rapidly changing, dynamic field of genomics. A number of issues were raised regarding aspects of a screening program including the purpose, benefits and harms, target population, program acceptability, components of a program, and economic evaluation.

#### Clarity of Purpose and Expected Benefits

Participants asserted that it would be important for governments to consider why preconception expanded carrier screening might be implemented as a publicly funded program, and what would be the objectives, motives, rationale, and goals of such a program. In their view, the purpose should be “well framed” with appropriate evidence of benefits and harms and ethical principles (e.g., autonomy and individual rights to informed healthcare choices and to make decisions with as much relevant information as possible). Discussions around the purpose and possible benefits of preconception expanded carrier screening focused on outcomes that might eventuate as a result of program participation. There were two clear perspectives on the overarching purpose or benefits, namely:

- Increased autonomy through increased reproductive choices: the information obtained through preconception expanded carrier screening leads to knowledge of carrier status and this increases the range of reproductive choices, to include not having a child with the conditions screened, or
- Reduced burden of disease: preconception expanded carrier screening could reduce infant mortality and morbidity. This is when identified carriers use the information on their carrier status to make reproductive choices that lead to the prevention or avoidance of children being born with conditions that are “life-threatening,” “severe,” “serious,” “nasty,” and “devastating” and with onset during childhood. There was the belief that this would result in fewer sick children being born.

There were tensions between some participants, who held differing views on whether a reduction in disease burden should be a primary goal or a secondary benefit of preconception expanded carrier screening.

Other potential benefits identified by participants included:

- Reduced family burden: the avoidance of births of affected children was linked to the belief that this would result in less distress, anxiety, strain, trauma, suffering, and long-term effects on families, which was then perceived to improve family quality of life. Participants related reduced burden of disease and family burden to the ethical principles of beneficence and prevention of harm.
- Equity of access: this refers to the notion that everyone in the target population should have access to all aspects of a screening program, including the screening test as well as information provided prior to screening. It was suggested that a government funded program for the whole population would mean less likelihood of a user-pays system. This would enable lower socioeconomic and vulnerable populations to access the program, thereby minimizing health disparities and inequities in access. However, there was some doubt expressed as to whether a preconception expanded carrier screening program would really have the capacity to deliver equitable access.
- Economic value: preconception expanded carrier screening could reduce healthcare expenditure through reducing morbidity and the subsequent need for lifetime care of people who are severely affected by the conditions screened.
- Consumer desire for health information: in making information on carrier status available for those who want it, preconception expanded carrier screening could be beneficial for those people who want as much information as possible related to their health.
- Increased genetics awareness among the public and health professionals: offering a preconception expanded carrier screening program to the whole population could empower people by increasing their knowledge about genetics (genetics literacy) and the fact that everybody is a carrier of something.

#### Potential Harms

There was a view that preconception expanded carrier screening may increase stigma and discrimination for those identified as carriers, those who opt not to undergo screening and those born with the conditions screened. It was also thought that people living with the conditions screened may be disadvantaged if a reduced incidence of these conditions reduces the incentives to develop treatments and therapies. According to participants, the rights of those who choose not to undergo preconception expanded carrier screening need to be respected. Further, participants felt that there would need to be adequate support for people regardless of the reproductive choice they make following preconception expanded carrier screening.

Participants argued that being identified as a carrier might have financial implications, for example, on insurance premiums, and psychological impacts, such as increased anxiety or false expectations or reassurance that they have been “promised” a healthy baby. Additionally, it was suggested that a government-sponsored preconception expanded carrier screening program might foster the perception of genetic testing being “routine” and that screening is mandated by the government, and thus not voluntary. People may feel social pressure, coercion, or obligation to participate. It might raise questions of government-sponsored eugenics and “where will it end?” A challenge was seen to be providing information and counseling that is “neutral,” particularly if there is a “strong incentive” to increase uptake to justify providing the program.

#### Target Population

The key issues explored by participants included defining the target population, deciding at what age to offer screening, and determining whether screening would be offered to both members of a couple at the same time or to one member of a couple first and only to the other if the first one is a carrier. There was also a perceived need for governments to understand what would motivate or drive decision-making around participation in a preconception expanded carrier screening program. Some attendees reflected that the uptake rate for the program has implications for cost-effectiveness, and the extent to which the program could result in benefits such as reduced burden of disease and increased reproductive choice.

#### Acceptability

Whether preconception expanded carrier screening was “acceptable” was raised by a number of participants. This included whether the general public and target population actually want government funded access to preconception expanded carrier screening, and whether clinicians and politicians would support such a program.

#### Components of a Program

Participants thought that, if a preconception expanded carrier screening program was to be offered by governments, there should be sufficient resources to invest in an “end to end service.” That is, the participants thought a program is not just about the screening test itself. Other components of a program that participants thought important for governments to consider included:

- The provision of information and education to the public, target population, and health professionals. Participants recommended that, as part of a program, information should be provided that would make the aim(s) of the program clear and encapsulate the benefits, risks, harms, consequences, uncertainties around genetics and preconception expanded carrier screening, and impact on individuals and society. Further, there was a view that program information should also outline the different conditions screened, testing procedure, interpretation of results, and implications. Some workshop participants were concerned that the public might not have the genetic literacy required to understand the information provided including the implications of results, and therefore would be faced with “information burden” or making reproductive decisions based on information that is poorly understood by themselves and/or health professionals. This raised a question around whether preconception expanded carrier screening would actually result in greater autonomy for couples wishing to make reproductive decisions.
- Informed consent. Information provision was linked to being able to provide informed consent to participate in a program. Questions were raised around what is the best way to realize informed consent and whether informed consent was possible if multiple conditions were offered for testing in the program. Linked to the need for informed consent was the need to provide pretest genetic counseling. Workshop participants also thought that posttest genetic counseling was essential to help program participants understand the carrier information they received and make informed decisions about what to do with the information they received.
- Clear care pathways and support for people identified as carriers. This included follow-up care in terms of enabling the reproductive choices that carriers might want to pursue (e.g., access to IVF or PGD). It was proposed that consideration would need to be given to how the preconception expanded carrier screening program would connect with these other services and what the implications of the program would be for other parts of the health system. According to participants, a preconception expanded carrier screening program needs to be integrated with other programs so that there are no mixed messages and quality is not compromised.
- Collection of data on program participants and program operations. There were concerns around participant privacy and data ownership, protection, confidentiality, sharing, and access.

Questions were also raised by participants around workforce capacity and the impact that a program may have on healthcare providers, how best to start the program, and whether a pilot study would be appropriate.

#### Economic Evaluation

The resources required to establish a high-quality “end to end” preconception expanded carrier screening program would likely be significant. Participants acknowledged that healthcare systems are experiencing both growing demand and funding ceilings. Consequently they argued there is a need for governments to prioritize spending and consider the opportunity costs of offering a preconception expanded carrier screening program, as opposed to any other program. It was thought that the establishment of a preconception expanded carrier screening program should not take resources away from providing adequate treatment of people who are living with the conditions screened.

There was a perceived need for governments to consider sustainability, cost–benefits, and cost-effectiveness, including direct, indirect, and intangible costs such as anxiety and other psychological harms. However, in making these suggestions, questions arose around how to best do this and what costs and savings should be considered and how can these be measured. In particular, participants questioned the best way to consider savings from reduced births of affected children and reduced long-term support for people living with severe disabilities. Government inertia and the difficulty of estimating costs were seen as inhibitors to investment in a preconception expanded carrier screening program.

## Discussion

Workshop findings highlight that there is a wide range of issues that require careful examination by governments that are assessing preconception carrier screening, to ensure that the benefits outweigh the harms. Overlaying feedback from the workshop against the original Wilson and Jungner principles demonstrates that these are not a “best fit” for governments to assess preconception expanded carrier screening. Given that only Israel has implemented a national program of genetic carrier screening (25) and only the Netherlands has developed tailored decision-making criteria for genetic screening, governments across the globe have further work ahead of them to develop criteria that could inform whether to introduce preconception expanded carrier screening. The workshop findings provide a starting point for governments to begin addressing this policy gap. Specifically, a range of issues have been identified in relation to the conditions to be screened, the tests to be used, and the components that should be incorporated into a preconception carrier screening program.

When considering “the condition,” workshop participants agreed that screening should only ever be offered for conditions that are “severe” or “serious.” This aligns with the Wilson and Jungner concept of an “important health problem” (12). However, participants recognized that there is no clear, consensus definition of what constitutes “severe,” with different suggestions existing in the literature (26, 27). Without a clear definition, it is difficult to determine the scope of conditions that should be considered for inclusion in an expanded screening program. Indeed there is marked disparity in the composition of currently available laboratory panels of conditions for expanded carrier screening (28). From a program perspective, a definition is essential because the number and type of conditions screened has follow-on implications for how the program is implemented. Specifically, it will impact upon components of the program such as information and consent requirements, as well as counseling requirements and treatment or follow-up options. Further, as outlined by workshop participants, the definition of “severe” and thus the conditions screened are likely to impact upon public and clinical perceptions of the program. If a clear definition is not developed, and parameters and safeguards not set, there is the potential for trust in a preconception expanded carrier screening program to be undermined. Therefore, a body of work is needed to consider the definition of “severe.” The definition offered by workshop participants is a valid starting point: “early onset conditions where the child dies in the newborn or early childhood period.” In excluding conditions that do not result in early mortality, this definition was perceived to be less ethically contentious and to have less of an impact on disability rights.

The workshop discussions around the Wilson and Jungner criteria for “the test” were aligned with the literature reviewed, in terms of the need for the test to be accurate (see Table 2). This was in relation to both sensitivity (low false-positives) and specificity (low false-negatives) and also the ability to determine meaningful residual risk for individuals who test negative. The issue of the cost-effectiveness of the tests was also raised by workshop participants, and this would likely be a key consideration for governments within the context of the overall cost-effectiveness of a program.

**Table 2:** **Coverage of issues referred to in literature**.

Topic area	Issue raised by workshop participants	Issue *not* raised by workshop participants
Condition	– Should be clinically severe (1, 5, 22, 23, 29, 30)	– The impact of the condition on individuals, families, and society needs to be understood (22) – It should be an important health problem (20) – The nature of the condition should influence the reproductive choices made by at-risk participants (19) – Conditions should have a predictable course (23) – The gene mutations that cause the condition should be understood, should have a valid clinical association with the phenotype/severity of the condition, and involve highly penetrant recessive inheritance (5, 7, 19, 29) – There should be a high frequency of carriers in the population (30–32)
Test	– Need to assess test performance and accuracy across all gene mutations assayed (5, 29, 32) – Sensitivity (1, 5, 23, 29, 30, 32) – Specificity (30, 32) – Ability to determine meaningful residual risk for individuals who test negative (7, 19, 33) – Cost-effective (29, 32)	– Non-invasive (32) – Accessible (31) – Straightforward interpretation of results (23) – Highly scalable to avoid limits to universal uptake (5, 32) – Limited to gene mutations that have the highest likelihood of being/are clearly pathogenic (1, 7) – Comply with laboratory guidelines that include quality control (19) – Inexpensive (5, 29, 32)
Treatment		– An effective treatment or intervention should be available for those identified as carriers (5, 20, 23)
Screening program	– A clearly defined purpose and benefits of carrier screening (1, 6, 21, 23, 32, 34) – Understanding of the potential harms of carrier screening, including physical, psychological, psychosocial, social, and ethical, which should all be low compared to benefits (20, 22, 35) – Equity and accessibility (29, 33) – Defining the target population and understanding their needs (23, 29, 36) – Reaching, inviting, recruiting, and informing the target population, and informing and educating the general public, acknowledging that both groups probably having low awareness or knowledge of carrier screening and the diseases screened for (21, 33, 37, 38) – Age to offer and timing of screening (i.e., individuals or couples) (1, 7, 21) – Participation that is voluntary and based on informed consent (1, 7, 20) – Consent processes and the provision of information, education, counseling, and support pre-screen for all participants and post-screen for those with positive test results, particularly about benefits and harms of screening, the test process, possible outcomes, interpretations of results, implications, and management options (1, 7, 20, 29, 30, 33, 34, 38–41) – Education of healthcare providers (1, 7, 21) – Resources and infrastructure, including access to follow-up services such as preimplantation genetic diagnosis and prenatal diagnosis (29, 30, 36, 42) – Public, political, and cultural attitudes toward and acceptability of carrier screening (1, 7, 33, 36) – Economic evaluations, particularly of cost–benefit and cost-effectiveness (1, 7, 21, 22, 32, 34) – Whether the program has been offered in the research setting and/or pilot studies and the outcomes of these studies (22, 31, 42)	– Accredited institutions and appropriately trained professionals (1) – Structure of the healthcare system (public/private) and implications for screening (43) – Monitoring, evaluation/review (21, 43, 44)

Workshop participants did not raise any considerations for government in relation to Wilson and Jungner’s theme of “the treatment.” This demonstrates a lack of salience of this issue for the participants. This could be because care and follow-up for carrier screening does not meet the traditional definition of treatment, since such screening does not result in the identification of people who have conditions. When coupled with the fact that much of the workshop discussion focused on elements of the screening program, the absence of discussion on treatment could also reflect heightened interest in the issue of “how to screen” as opposed to “whether to screen.” Nonetheless, the relevance of the Wilson and Jungner criteria associated with “the treatment” may be queried in relation to preconception expanded carrier screening. The question then becomes whether there are more relevant dimensions that should replace the treatment criterion. For example, instead of the need for treatment being available, should the criterion be to recommend that “interventions are available”? Should the issue of interventions be framed within the context of the reasons for participation in preconception expanded carrier screening, such as “a decision should need to be taken by the person screened” (20) or that “screening should potentially influence the reproductive choices made by at-risk participants” (19)?

In our view, it is essential that governments consider the availability of interventions for preconception expanded carrier screening, and the downstream effects on and costs of providing such interventions. In order for a screening program to be effective and cost-effective, there must be an intervention that can lead to better health outcomes for an individual. Further, the intervention must be effective, available, easily accessible, and acceptable to individuals within the target population (44). Importantly, government consideration should be given to the fact that interventions for individuals identified as carriers are not currently always equitably accessible. For example, IVF and PGD are provided in the private sector within Australia, meaning there can be significant costs to individuals, which may limit access for citizens in lower socioeconomic groups (45, 46). This means that Australia, and other countries where these healthcare services are not equitably accessible, would need to carefully consider its capacity to provide the follow-up interventions required for a population-based approach to preconception expanded carrier screening. Further in relation to equity, consideration would also need to be given to the quality of PGD and IVF services, particularly given concerns regarding false-positive screening results (47, 48), and the fact that the genetics workforce is not keeping pace with the demand for these services (49).

As with the review by Seedat et al. (14) of population-based screening criteria adopted across a number of countries, the findings of the workshop were more likely to focus on considerations relating to “the screening program” as opposed to the condition, test, and treatment. The issues identified in relation to the screening program were largely those that would be relevant to all screening programs, not only preconception expanded carrier screening. These issues included the need for a program that is not just about the test, but rather includes components such as the provision of information and education, informed consent processes, genetic counseling, clear care pathways, data collection, and economic evaluation. There is a need for further exploration of these issues to determine in what ways, if any, these program components would be impacted by the unique features of expanded carrier screening. For example, there was recognition by workshop participants that consent should be informed, but what would be the impact on the ability to obtain informed consent, when expanded carrier screening would test for multiple conditions simultaneously? How would informed consent be defined in this context? Related to this issue, further investigation should examine the impact of preconception expanded carrier screening on the complexity, volume, and financial implications of pretest and posttest counseling (28).

In addition to the work that is needed by governments to develop robust decision-making models for assessing preconception expanded carrier screening, researchers should begin to explore a number of issues raised by the workshop participants to inform and complement work in the public policy space. Within local contexts, “societal readiness” for preconception expanded carrier screening could be investigated. While several potential benefits of expanded carrier screening were identified at the workshop, a number of potential harms were also discussed, including concerns around discrimination, eugenics, and people refusing to participate in a program, which could undermine the cost-effectiveness of program delivery. While the UK Human Genetics Commission (18) concluded “there are no specific ethical, legal or social principles that would make preconception genetic testing within the framework of a population screening program unacceptable” (p. 1), this needs to be explored by experts in other local contexts, including the contention expressed by workshop participants around the primary purpose of this screening being reproductive choice and/or reduced burden of disease (50). Further to this, consultation and engagement methodologies could be developed and implemented to assess stakeholder acceptability of preconception expanded carrier screening, including the public, target population, disease associations, clinicians, and laboratory staff. For the target population, this should also include investigation of likely uptake and postscreening decisions around reproductive choices. A recent study in the Netherlands has made initial contributions in the area of citizens/user perceptions of expanded carrier screening (51), while a qualitative study in Sweden has examined healthcare professionals’ views on preconception carrier screening (52). This line of work must be extended to further local contexts.

This paper has several limitations. Workshop participants were self-selected and may not be a representative sample of experts relevant to preconception expanded carrier screening. This may impact on the generalizability of the workshop findings. It is also important to note that, in relation to the literature on existing carrier screening programs and recommendations by professional bodies regarding expanded carrier screening, not all of the issues raised in the literature as key success factors or recommendations for implementation were addressed by the participants (see Table 2). During the workshop, participants were exposed to a range of perspectives related to preconception expanded carrier screening, which framed the subsequent discussions, and not all perspectives were covered by the workshop presentations. Participant exposure to other perspectives may have resulted in different workshop outcomes. Finally, as noted above, the workshop findings were reasonably high-level and did not drill down to deeper levels of analysis regarding the key issues. Therefore, while the findings provide useful guidance, a more precise exploration of each issue may be required to develop a comprehensive view of the factors governments need to consider when deciding whether to implement preconception expanded carrier screening.

The international workshop was an important opportunity for expert stakeholders in the field of preconception expanded carrier screening to come together to share their values, experiences and knowledge. The workshop outcomes identified benefits, harms, and other key issues that governments should consider when assessing whether to publicly fund preconception expanded carrier screening programs. This is particularly useful since most countries globally do not have decision-making frameworks related to emerging genetic screening options and are at the formative stage of making assessments about preconception expanded carrier screening.

## Author Contributions

All the authors contributed substantially to the conception and design of the workshop, the acquisition and interpretation of data, have given final approval for the manuscript to be published, and agreed to be accountable for all aspects of the work. CM and KL undertook the data analysis and drafted the manuscript. GB, SM-J, AC, VS, HD, and NL critically revised the manuscript for important intellectual content.

## Conflict of Interest Statement

The authors acknowledge the financial contribution of Life Letters to the implementation of the workshop reported in this paper.
